# Clinical relevance of thyroid cell models in redox research

**DOI:** 10.1186/s12935-015-0264-3

**Published:** 2015-12-09

**Authors:** Francesca Cammarota, Francesco Fiscardi, Tiziana Esposito, Gabriella de Vita, Marco Salvatore, Mikko O. Laukkanen

**Affiliations:** IRCCS SDN, Via E. Gianturco 113, 80143 Naples, Italy; Department of Molecular Medicine and Medical Biotechnologies, University of Naples Federico II, 80014 Naples, Italy

**Keywords:** Thyroid cancer, Redox gene expression, Cell line, Cell model, Rat, PC Cl3, FRTL5, SOD3, Extracellular superoxide dismutase

## Abstract

**Background:**

Thyroid-derived cell models are commonly used to investigate the characteristics of thyroid cancers. It is noteworthy that each in vitro single cell model system imitates only a few characteristics of thyroid cancer depending on e.g. source of cells or oncogene used to transform the cells.

**Methods:**

In the current work we utilized rat thyroid cancer cell models to determine their clinical relevance in redox gene studies by comparing in vitro expression data to thyroid *Oncomine* microarray database. To survey the cell lines we analyzed mRNA expression of genes that produce superoxide anion (*nox* family), genes that catalyze destruction of superoxide anion to hydrogen peroxide (*sod* family), and genes that remove hydrogen peroxide from cellular environment (*catalase*, *gpx* family and *prdx* family).

**Results:**

Based on the current results, rat thyroid PC Cl3, PC PTC1, PC E1A, or FRLT5 cell models can be used to study *NOX2*, *NOX4*, *SOD2*, *SOD3*, *CATALASE*, *GPX1*, *GPX2*, *GPX5*, *PRDX2*, and *PRDX3* gene expression and function.

**Conclusions:**

Redox gene expression in rat originated single cell model systems used to study human thyroid carcinogenesis corresponds only partly with human redox gene expression, which may be caused by differences in redox gene activation stimulus. The data suggest careful estimation of the data observed in rat thyroid in vitro models.

**Electronic supplementary material:**

The online version of this article (doi:10.1186/s12935-015-0264-3) contains supplementary material, which is available to authorized users.

## Background

Tumors of thyroid gland, which include e.g. differentiated papillary thyroid cancer and poorly differentiated anaplastic thyroid cancer, are among the foremost-characterized cancers. A number of cell models have been created to study signal transduction and function of oncogenes in thyroid carcinogenesis [1–4]. These model systems, however, may not reflect all aspects of thyroid cancers emphasizing the need to compare the gene expression between the cell model system and human tissues.

Although the function of reactive oxygen species (ROS) is unspecific they are important mediators of tumor formation inducing mutations and regulating signal transduction, thus affecting a large range of cellular molecules. Reduction/oxidation (redox) system is highly sensitive to cellular changes, such as ageing and transformation, and therefore can function as a sensor in cell differentiation and carcinogenesis. The importance of ROS for primary cell immortalization and transformation was emphasized by a study demonstrating that increased *nox1* expression is essential for cellular transformation by *ras* oncogene [5]. NOX proteins are catalytic subunits in NADPH complexes inducing superoxide anion (O_2_^−^) production and activating signal transduction by interacting with other cellular proteins. Superoxide dismutase (SOD) gene family catalyzes dismutation of superoxide anion into hydrogen peroxide (H_2_O_2_), which is then further metabolized by catalase, glutathione peroxidase (GPX) family, and peroxiredoxin (PRDX) family.

To characterize thyroid cancer models derived from rat primary thyroid cells we analyzed the expression of different *nox* genes representing superoxide anion radical source and *sod*, *catalase*, *gpx*, and *prdx* gene families that dismutase superoxide to hydrogen peroxide and further to less reactive derivatives, such as oxygen and water molecules. The obtained expression data was compared to microarray data from thyroid cancer patients to validate the use of cell models in redox studies.

## Results

### Expression of *NOX1*-*4*, *SOD1*-*3*, *GPX1*-*8*, and *PRDX1*-*6* genes in normal human thyroid, papillary thyroid cancer, and in anaplastic thyroid cancer

**Fig. 1:**
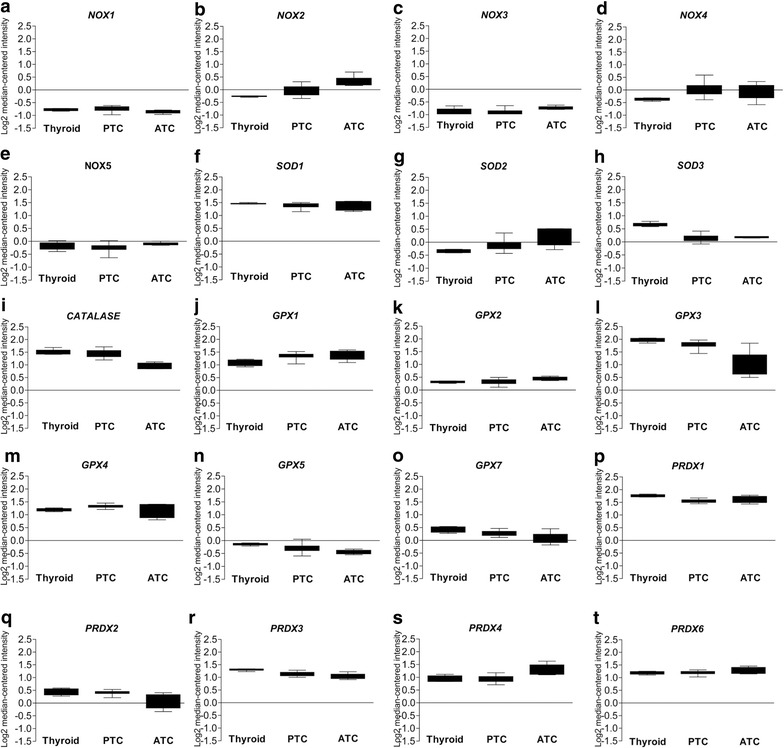
Microarray data extracted from Oncomine database. The average expression of redox genes obtained from the database is shown as a histograms. **a** expression of *nox1*, **b** expression of *nox2*, **c** expression of *nox3*, **d** expression of *nox4*, **e** expression of *nox5*, **f** expression of *sod1*, **g** expression of *sod2*, **h** expression of *sod3*, **i** expression of *catalase*, **j** expression of *gpx1*, **k** expression of *gpx2*, **l** expression of *gpx3*, **m** expression of *gpx4*, **n** expression of *gpx5*, **o** expression of *gpx7*, **p** expression of *prdx1*, **q** expression of *prdx2*, **r** expression of *prdx3*, **s** expression of *prdx4*, **t** expression of *prdx6*

Although ROS are important second messengers in normal cellular functions, in pathological conditions, such as cancer, redox enzyme expression is unbalanced causing oxidative stress. Thyroid carcinogenesis models consist of a number of different in vitro models that are derived from rat origin by transforming the cells with oncogenes. Since rat and human thyroid models may have different characteristics, especially in highly sensitive redox system, in the current work we analyzed redox gene expression in PC Cl3 and FRLT5 derived thyroid cancer models. For the survey we selected redox enzymes producing superoxide anion (O_2_^−^) and on enzymes neutralizing it to hydrogen peroxide (H_2_O_2_) and further to e.g. water (H_2_O) and oxygen (O_2_). To compare the observed redox gene expression in rat thyroid cell models with human thyroid tissue and thyroid cancers we first extracted microarray data from Oncomine database (http://www.oncomine.org) that contains a number of patients and hence moderate the differences observed between individuals. *NOX1*, *NOX3*, and *NOX5* expression suggested minor changes in thyroid cancers (Fig. 1a, c, e), whereas the expression of *NOX2* and *NOX4* showed increased mRNA synthesis in papillary thyroid and anaplastic thyroid cancers (Fig. 1b, d). *SOD1* stayed at similar levels, *SOD2* expression increased in cancer, and *SOD3* mRNA synthesis decreased correlating to reduced differentiation degree of thyroid tissue (Fig. 1f–h). *CATALASE* expression was markedly decreased in anaplastic thyroid cancer as compared to normal thyroid tissue and papillary thyroid cancer (Fig. 1i). Glutathione peroxidase family showed variable gene expression: *GPX1* and *GPX2* expression analysis suggested minor increase in mRNA synthesis (Fig. 1j, k), *GPX3*, *GPX5*, and *GPX7* expression was downregulated in thyroid cancers (Fig. 1l, n, o), whereas *GPX4* expression did not change (Fig. 1m). *PRDX1*, *PRDX2*, and *PRDX3* mRNA synthesis decreased in papillary and anaplastic thyroid cancers (Fig. 1p–r), PRDX4 mRNA expression was moderately increased in anaplastic thyroid cancer tissues (Fig. 1s), and *PRDX6* expression status was at similar levels in normal thyroid and thyroid cancers (Fig. 1t).

### TSH increases redox gene expression in rat FRLT5 cells

Rat thyroid cells immortalized and transformed with oncogenes are widely used to study the development of thyroid cancer. Previous studies have suggested increased ROS production in cells treated with thyroid stimulating hormone (TSH) [6, 7], which binds to G-protein couple TSH receptor (TSH-R) and activates downstream signal cascades, such as cyclic AMP, PKA, PKC, ERK1/2, PI3K-AKT. Hence, we dissected *nox1*-*4*, *sod1*-*3*, *catalase,gpx1*-*8*, and *prdx1*-*6* expression status in rat thyroid FRLT5 stimulated with TSH.

**Fig. 2:**
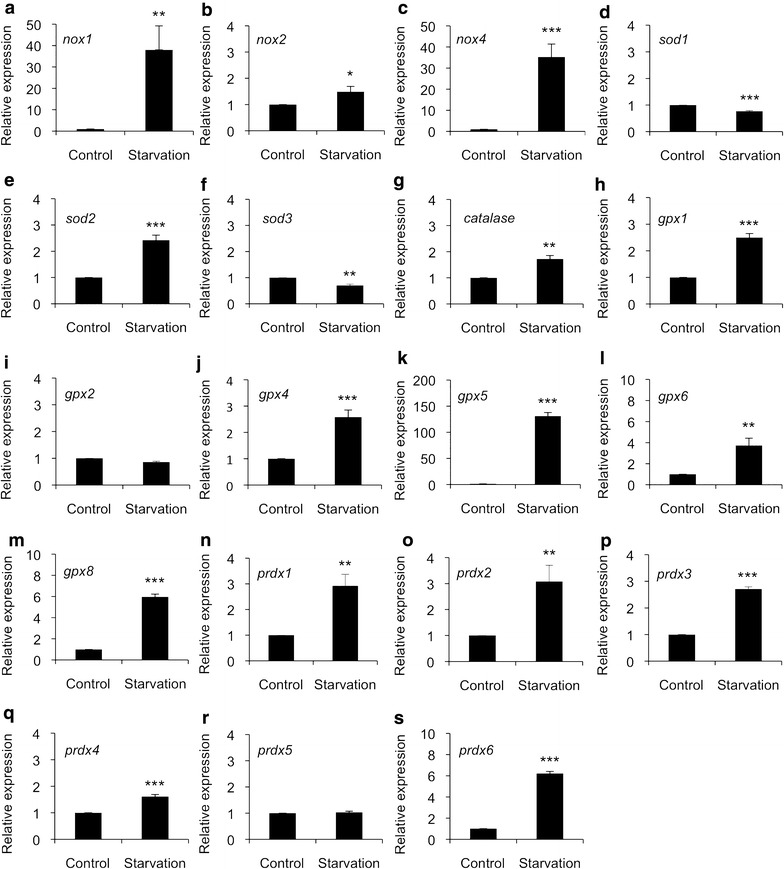
The effect of TSH on redox gene expression. FRLT5 cells were treated with TSH after 3-day hormone starvation. **a** expression of *nox1*, **b** expression of *nox2*, **c** expression of* nox4*, **d **expression of *sod1*, **e** expression of *sod2*,** f **expression of *sod3*, **g** expression of *catalase*, **h** expression of *gpx1*,** i** expression of *gpx2*,** j** expression of *gpx4*, **k** expression of *gpx5*, **l **expression of *gpx6*, **m **expression of* gpx8*,** n **expression of *prdx1*, **o **expression of *prdx2*, **p **expression of *gpx3*,** q **expression of *prdx4*,** r** expression of *prdx5*,** s** expression of* prdx6*. The data are expressed as the mean ± SD. The p values are represented (*p < 0.05, **p < 0.01, ***p < 0.001)

The data shown in Fig. 2 suggested that TSH stimulation of cells after three-day hormone starvation induced mRNA production of *nox1*, *nox2*, and *nox4* genes (Fig. 2a–c), whereas *nox3* was not expressed or the expression level was extremely low and thus not detectable in FRLT5 thyroid cells. The expression analysis of superoxide dismutase family members, which dismutase O_2_^−^ to H_2_O_2_, suggested decreased cytoplasmic *sod1* and extracellular *sod3* mRNA synthesis, whereas the expression of mitochondrial *sod2* was increased (Fig. 2d–f). Interestingly, there was a significant upregulation of genes responsible of H_2_O_2_ removal; the expression of *catalase*, *gpx1*, *gpx4*, *gpx5*, *gpx6*, *gpx8*, *prdx1*, *prdx2*, *prdx3*, *prdx4*, and *prdx6* were all upregulated (Fig. 2g, h, j–q, s). Only *gpx2* and *prdx5* (Fig. 2i, r) did not respond to TSH starvation, and FRTL5 cells did not express *gpx3* and *gpx7*, or their expression level was extremely low.

### Effect of *PTC1* and *e1a* oncogenes on redox gene expression

**Fig. 3:**
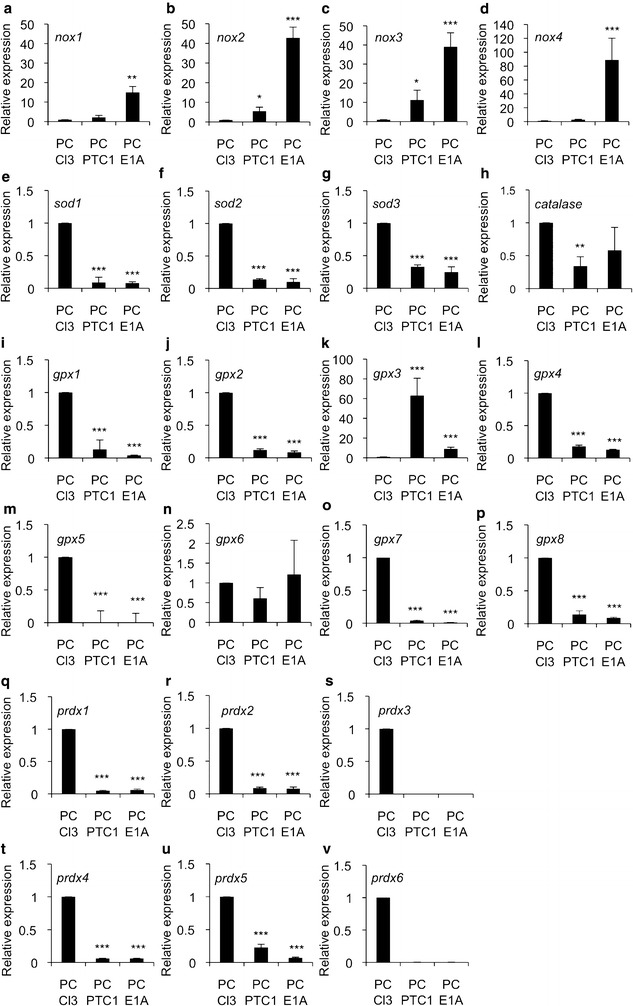
The effect of *PTC1* and *e1a* oncogenes on redox gene expression in PC Cl3 cell derived cancer models. **a** expression of *nox1*, **b** expression of *nox2*, **c** expression of *nox3*, **d** expression of *nox4*, **e** expression of *sod1*, **f** expression of *sod2*,** g** expression of *sod3*, **h** expression of *catalase*, **i** expression of *gpx1*,** j** expression of *gpx2*, **k** expression of *gpx3*,** l** expression of *gpx4*, **m** expression of *gpx5*, **n** expression of *gpx6*,** o **expression of *gpx7*, **p** expression of *gpx8*, **q** expression of *prdx1*, **r** expression of *prdx2*, **s** expression of *prdx3*,  **t** expression of *prdx4*, **u** expression of *prdx5*, **v** expression of *prdx6*. The data are expressed as the mean ± SD. The p values are represented (*p < 0.05, **p < 0.01, ***p < 0.001)

In cancer ROS, especially superoxide anion, mediates primary cell transformation and cell proliferation [5]. We thus analyzed the effect of *PTC1* and *e1a* oncogene expression on redox gene mRNA levels in rat PC Cl3, PC PTC1 and PC E1A cells. As shown in Fig. 3, the mRNA levels of *nox1*-*4* were upregulated by the oncogenes, although in PC PTC1 cells there was only a tendency for increased *nox1* and *nox4* mRNA expression. Interestingly, while O_2_^−.^ production was upregulated, there was a downregulation of genes dismutating O_2_^−.^ to H_2_O_2_ and of genes removing H_2_O_2_ from the cellular environment. The expression of *sod1*-*3*, *cat, gpx1*, *gpx2*, *gpx4*, *gpx5,gpx7*, and *gpx8*, and *prdx1*-*6* genes was mostly decreased by *PTC1* and *e1a* oncogenes thus suggesting redox unbalance towards increased O_2_^−^ cellular concentration. The expression analysis suggested reduced, or tendency towards reduced *catalase* and *gpx6* expression in *PTC1* transformed PC Cl3 cells but not clear effect in *e1a* transformed cells. Only the expression of *gpx3* was upregulated by *PTC1* and *e1a* oncogenes. Based on the data there is increased expression of *nox* genes forming NADPH complex that mainly produces O_2_^−^, whereas expression of genes coding for enzymes responsible for removing ROS was mostly downregulated.

### The effect of RAS on redox gene expression

**Fig. 4:**
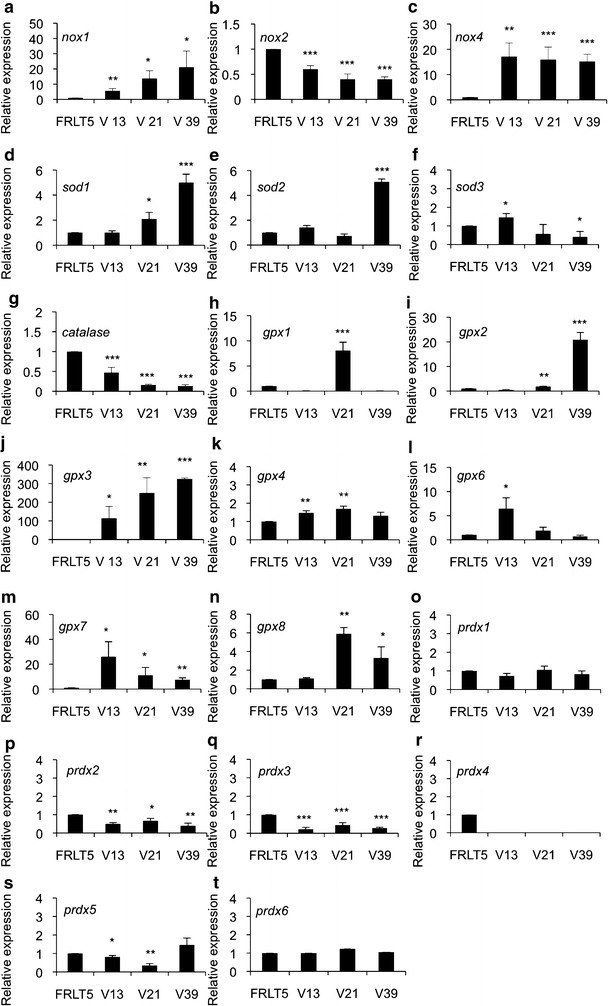
The effect of *H*-*RasV12* oncogene on redox gene expression in FRLT5 cells derived cancer model. **a** expression of *nox1*,** b** expression of *nox2*, **c **expression of *nox4*,** d** expression of *sod1*,** e** expression of *sod2*,** f** expression of *sod3*, **g** expression of *catalase*,** h** expression of *gpx1*,** i **expression of *gpx2*, **j** expression of *gpx3*, **k** expression of *gpx4*,** l** expression of *gpx6*, **m **expression of* gpx7*, **n **expression of *gpx8*, **o** expression of *prdx1*, **p** expression of* gpx2*, **q** expression of *prdx3*, **r **expression of* prdx4*,** s** expression of *prdx5*, ** t** expression of *prdx6*. The data are expressed as the mean ± SD. The p values are represented (*p < 0.05, **p < 0.01, ***p < 0.001)

Next we investigated the expression of redox genes in FRLT5 cell model that consists of cells harboring different levels of RAS: FRLT5 clone V13 has 1.4-fold increased RAS activation, clone V21 has tenfold increased RAS activation, and clone V39 has 35-fold increased RAS activation as compared to FRLT5 control cells [2]. The expression analysis of *nox* gene family suggested gradually increased *nox1* mRNA synthesis and decreased *nox*2 expression correlating to increased RAS activation level (Fig. 4a, b), whereas *nox4* expression was strongly upregulated in FRLT5 V13, FRLT5 V21, FRLT5 V21, and in FRLT5 V39 clones as compared to control FRLT5 cells (Fig. 4c). In line with previous data [8], *sod1* and *sod2* expression varies depending on the cell model used. Gradually increased RAS activation correlated to increased *sod1* mRNA levels whereas *sod2* synthesis suggested differential pattern (Fig. 4d, e). The expression analysis of *sod3* showed increase in mRNA synthesis at low RAS activation levels and significantly decreased *sod3* mRNA production at high RAS levels (Fig. 4f), as we have published recently [8]. Similarly with dismutase family, genes responsible of H_2_O_2_ removal showed variable expression. *Catalase* expression was gradually downregulated (Fig. 4g), whereas gene expression analysis of members of glutathione peroxidase family suggested increased mRNA synthesis. *Gpx1* and *gpx6* mRNA expression was upregulated in clone V21 (tenfold RAS activity) and in clone V13 (1.4-fold RAS activity), respectively (Fig. 4h, l). Other members of *gpx* gene family, *gpx2*, *gpx3*, *gpx4*, *gpx7*, and *gpx8* showed increased mRNA production corresponding to increased RAS activity (Fig. 4i–k, m, n). The expression of *peroxiredoxin* family members was decreased already at moderate 1.4-fold RAS activation level excluding *prdx1* and *prdx6* that showed only a minor changes in mRNA synthesis levels in the cell model (Fig. 4o–t).

### TPA has a time-dependent effect on redox gene expression

**Fig. 5:**
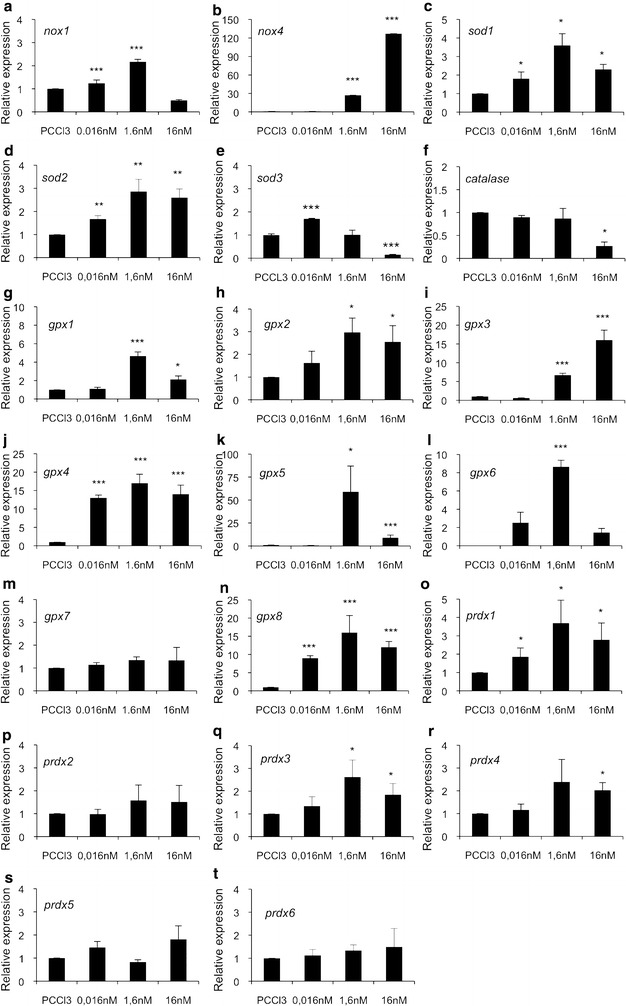
Dose-dependent effect of TPA on redox gene expression in PC Cl3 cells.** a** expression of *nox1*, **b** expression of *nox4*, **c** expression of *sod1*, **d** expression of *sod2*, **e** expression of *sod3*, **f** expression of* catalase*, **g** expression of *gpx1*, **h** expression of *gpx2*,** i** expression of* gpx3*,** j** expression of *gpx4*, **k **expression of *gpx5*,** l** expression of *gpx6*,** m **expression of *gpx7*, **n **expression of *gpx8*, **o** expression of *prdx1*,** p** expression of *gpx2*,** q** expression of *prdx3*, **r **expression of *prdx4*,** s** expression of *prdx5*, ** t** expression of prdx6. The data are expressed as the mean ± SD. The p values are represented (*p < 0.05, **p < 0.01, ***p < 0.001)

The gene expression analysis of PC Cl3 cells treated with 1.6 nM tetradecanoylphorbol acetate (TPA) on three consecutive days was studied to dissect the effect of general mitogenic pressure on redox gene expression. There was only a modest increase in *nox1* expression and a significant long-term increase in *nox4* expression (Fig. 5a, b). The expression of *nox2* and *nox3* mRNA synthesis was not detected suggesting that TPA treatment decreased their mRNA synthesis unlike *PTC1* and *e1a* oncogenes (Fig. 3b, c). The expression of cytoplasmic *sod1* and mitochondrial *sod2* was significantly increased at all time points, whereas extracellular *sod3* mRNA production showed modest reduction in gene expression during the treatment (Fig. 5c–e). Cytoplasmic *catalase* expression was significantly increased on all three consecutive days (Fig. 5f). The overall pattern of *glutathione peroxidase* gene family mRNA synthesis suggested initially increased expression that returned towards the baseline on day three. *Gpx1* expression was significantly increased on day 1 and on day 2, whereas on day 3 there was only an insignificant tendency for increased mRNA synthesis (Fig. 5g). The gene expressions of *gpx2*, *gpx3*, *gpx4*, *gpx5*, *gpx6*, and *gpx8* were significantly increased during the treatment (Fig. 5h–l, n), whereas the mRNA synthesis of *gpx7* was increased only on day 2 and returned back to the base line on day 3 (Fig. 5m). *Prdx 1*-*6* gene expressions were upregulated by TPA treatment on all consecutive days (Fig. 5o–t).

### TPA has a concentration dependent effect on redox gene expression

**Fig. 6:**
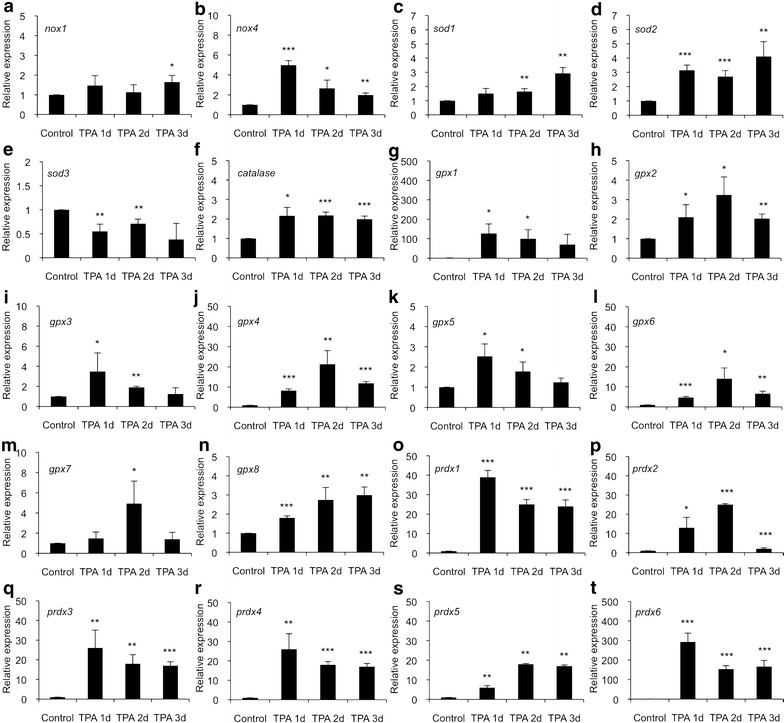
Time-dependent effect of TPA on redox gene expression in PC Cl3 cells. **a** expression of *nox1*, **b** expression of *nox4*, **c** expression of *sod1*, **d** expression of *sod2*, **e **expression of *sod3*,** f** expression of *catalase*, **g **expression of *gpx1*, **h** expression of *gpx2*,** i **expression of* gpx3*,** j** expression of *gpx4*,** k** expression of *gpx5*, **l** expression of *gpx6*, **m** expression of *gpx7*, **n** expression of *gpx8*,** o** expression of *prdx1*, **p** expression of* gpx2*, **q **expression of *prdx3*, **r **expression of* prdx4*, **s** expression of *prdx5*, ** t** expression of *prdx6*. The data are expressed as the mean ± SD. The p values are represented (*p < 0.05, **p < 0.01, ***p < 0.001)

To further dissect the effect of mitogenic pressure on redox gene expression we treated rat thyroid PC Cl3 cells with 0.016, 1.6, and 16 nM TPA. We observed increased *nox1* expression at 0.016 nM dose that was further strengthened at 1.6 nM concentration and returned back to baseline at high 16 nM TPA (Fig. 6a), whereas *nox4* expression initiated to increase at 1.6 nM concentration and suggested robust mRNA production at 16 nM TPA (Fig. 6b). The expression of *nox2* and *nox3* was not detectable supporting the data shown in Fig. 4. *Sod1* and *sod2* mRNA expression analysis suggested significantly increased mRNA synthesis at all TPA doses used (Fig. 6c, d). *Sod3* expression analysis suggested significantly increased mRNA synthesis at low 0.016 nM TPA concentration, baseline expression at 1.6 nM TPA, and significantly decreased expression at high 16 nM TPA (Fig. 6e). *Catalase* expression was at similar levels with control cells at 0.016 nM and at 1.6 nM TPA doses, and decreased at 16 nM TPA (Fig. 6f). The expression analysis of *glutathione peroxidases* family members *gpx1*, *gpx2*, *gpx3*, *gpx4*, *gpx5*, *gpx6*, *gpx7*, and *gpx8* suggested increased mRNA synthesis correlating to increased TPA concentration (Fig. 6g–n). The expression analysis of *peroxiredoxins* suggested increased mRNA synthesis of *prdx1* at all TPA doses (Fig. 5o), of *prdx3* at 1.6 nM and at 16 nM TPA concentrations (Fig. 6q), and of *prdx4* at 16 nM TPA (Fig. 6r). The expressions of *prdx2*, *prdx5*, and *prdx6* were unaffected at all TPA concentrations (Fig. 6p, s, t).

### SOD3 enhances the TPA derived increased cell proliferation and loss of normal cellular phenotype

**Fig. 7:**
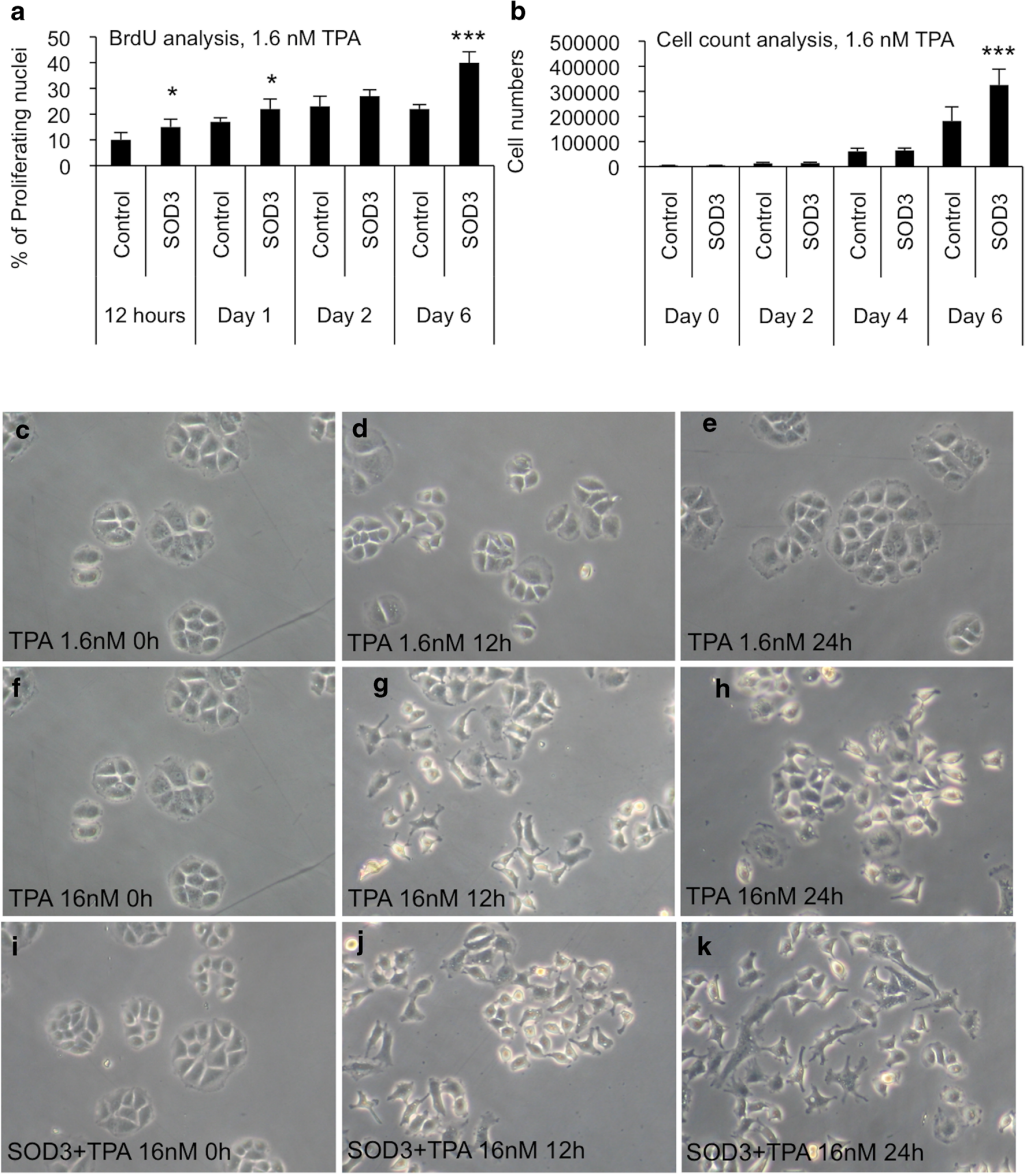
The effect of *SOD3* over-expression on TPA treated PC Cl3 cells. **a** BrdU incorporation analysis suggested *SOD3*-enhanced DNA replication. **b** Growth curve analysis supported BrdU assay suggesting increased growth in the presence of *SOD3*. **c**–**e** The effect of 1.6 nM TPA on PC Cl3 cell follicular growth formation. **f**–**h** The effect 16 nM TPA on PC Cl3 cell follicular growth formation. **i**–**k** The combined effect of 16 nM TPA and SOD3 over-expression on PC Cl3 cell follicular growth formation. The data are expressed as the mean ± SD. The p values are represented (*p < 0.05, **p < 0.01, ***p < 0.001)

Based on the current data *sod3* gene expression showed RAS activation-dependent and TPA concentration-dependent initial increase that was then decreased at high RAS activation and TPA concentration levels (Figs. 4f, 6e). Thus, we studied the effect of *SOD3* over-expression on the growth of PC Cl3 cells during TPA treatment. BrdU DNA incorporation and cell count analysis suggested that *SOD3* over-expression significantly enhanced TPA stimulated DNA proliferation and cell growth as compared to control cells (Fig. 7a, b). PC Cl3 cells characteristically grow in follicular clusters in the growth medium supplemented with thyroid hormones and growth factors. TPA treatment had a concentration and a time-dependent effect on control PC Cl3 cells causing the loss of the characteristic follicular cluster conformation phenotype (Fig. 7a–h). At 1.6 nM TPA concentration (Fig. 7a–c) the loss of the follicular growth formation was slower than at high 16 nM TPA concentration (Fig. 7f–h) that disrupted the normal growth phenotype already at 12-h time point after the initiation of TPA supplementation. PC Cl3 cells over-expressing human *SOD3* showed earlier loss of the follicular cluster conformation phenotype that initiated at 12-h time point and suggested spindle like phenotype at 24-h time point (Fig. 7i–k).

## Discussion

Primary cell transformation and malignant cancer development is frequently characterized by increased oxidative stress that induces mutations and increases growth signaling. The data extracted from *Oncomine* database suggested increased *NOX1*-*5* gene expression that, as part of NADPH oxidase complex, induces O_2_^−^ production (Fig. 1). In line with previously published reports, *Oncomine data* showed reduced expression of genes coding for enzymes that remove oxygen radicals from the tissue environment. The only exceptions were increased expression of mitochondrial SOD2, GPX1, and GPX2 [9–11] thus highlighting the importance of mitochondrial activity in tumorigenesis [12].

Thyroid stimulating hormone is an important mediator of normal thyroid physiology whereas transformed thyroid cells develop the ability to grow independently of hormone stimulation [13]. Several thyroid pathologies are characterized by abnormal TSH production thus compromising hormone related signal transduction and thyroid function. TSH acts through TSH receptor, a member of small G-protein coupled receptor family, regulating thyroid hormone T3 and T4 production, thyroid growth, and cell differentiation through adenylyl cyclase and cAMP signaling pathways [14]. TSH concentration is markedly increased in Hashimoto’s disease, which is also characterized by increased oxidative stress [15], whereas Graves’s disease is characterized by low levels of TSH in compensation of high T3 and T4 hormone concentrations in circulation. Grave’s disease is an autoimmune disease associated with increased oxidative stress, mainly originating from migrating inflammatory cells [16]. The current data showed TSH-stimulated signal transduction regulating redox gene expression in FRLT5 cells (Fig. 2) suggesting feasibility of the cells for Hashimoto’s disease and Grave’s disease related oxidative stress studies. In support of the current data, we have previously shown in line with the current work that TSH stimulation decreases *sod3* expression in PC Cl3 cells [6].

Thyroid cancers arise from epithelial cell mutations, such as *RET/PTC* genetic aberration formed by chromosomal rearrangement of *RET* tyrosine kinase domain to N-terminal of H4 (D10S170) [17] and frequently associated to radiation derived papillary thyroid cancer [18]. A number of cell lines expressing oncogenes, e.g. adenovirus adenovirus *Ad12early region 1A* (*e1a*), are used to study the development and characteristics of thyroid cancer. The oncogenic nature of *e1a* has been linked to virus dose, host genetic constitution, age of the host at the time of infection, and immune status of the host. The viral *e1a* sequence drives primary cells towards transformation by affecting signal transduction pathways involved in immune escape, p53 response, and protein stability [19]. Rat thyroid PC Cl3 cells transfected with *e1a* lack the differentiation ability and show characteristics of TSH independent growth and function [20]. The frequency of *ras* mutations in thyroid tumors vary from 0 % up to 85 % showing increased occurrence in poorly differentiated and undifferentiated thyroid cancers [21, 22]. The aberrant activation of RAS-BRAF-MEK1/2-ERK1/2 kinase signaling pathway, which mediates the extracellular signals into the nucleus, contributes to thyroid carcinoma by increasing cell proliferation, survival, and metastasis.

In the current work redox gene expression PC Cl3 cells transformed with *PTC1* and *e1a* oncogenes suggested partial similarity between human microarray data and cell models. The expression of *nox2*, *nox3*, and *nox4* in cell models showed similar increase as in thyroid cancers tissues whereas the decreased expression of *sod3*, *catalase*, *gpx5*, *gpx7*, *prdx1*, *prdx2*, and *prdx3* corresponded to decreased expression in microarray data. It is important to note that the expression of *nox1*, *sod1*, *sod2*, *gpx1*, *gpx3*, *gpx4*, *gpx8*, *prdx4*, and *prdx6* in cell models was opposite to human tissues. Further, *gpx6* and *prdx5* that showed variable expressions in cell models were not expressed in human cancers (Fig. 3). Thus, out of 22 redox genes 10 showed similar expression pattern in thyroid tissues and *PTC1* or *e1a* derived cell models. In FRLT5 based *H*-*RasV12* cell model the expression of *nox4*, *sod2*, *sod3*, *catalase*, *gpx2*, *gpx4*, and *prdx6* corresponded to human tissue mRNA production. The expression of *gpx1* was variable and the expression of *nox1*, *nox2*, *sod1*, *gpx3*, *gpx7*, *prdx1*, *prdx2*, *prdx3*, and *prdx4* in cell model was opposite to expression in thyroid tissue. In line with *PTC1* and *e1a* models, *gpx6*, *gpx8*, and *prdx5*, which were absent in human microarray data, showed mRNA production in FRLT5 cell model indicating that seven redox genes out of 22 had similar expression pattern in cells and tissues (Fig. 4).

The differences between tissue microarray database and in vitro gene expression assays may be caused by presence of stromal paracrine effect influencing tissue redox balance or presence of multiple genetic aberrations in vivo. In human tissues increased expression of *NOX* genes is mainly caused by small RAS GTPase protein activation [23–25] or cytokines stimulus [26–28], thus suggesting direct involvement of *ras* family genes in *nox* mRNA production in FRLT5 clones V13, V21, and V39 (Fig. 4). Similarly with *NOX* genes *ras* oncogene signaling has been suggested to be involved in *SOD1*, *SOD2*, and *SOD3* gene expression through PI3K-AKT [29, 30] and RAS-RAF-MEK-ERK pathways [31] *CATALASE* mRNA expression, which was decreased in vitro models by oncogenes, is stimulated by PI3K-AKT and AMPK signaling [32, 33], whereas glutathione peroxidase family gene expression, another H_2_O_2_ metabolizing enzyme group, is activated by proto-oncogenes, such as *ets*-*1* [34], by antioxidative *Keap1* signaling [35], by peroxisome proliferator-activated receptor γ signaling [36], by ROS [37], and by cytokines [35]. The third H_2_O_2_ metabolizing enzyme group, peroxiredoxin gene family, is activated by immune response inducing proteins, such as lipopolysaccharide through ROS-p38 MAPK [38, 39] by proto-oncogene SRC-PI3 K-JNK pathway [40], by PI3K-AKT [41], by cytokines [42, 43] and by various kinds of antioxidative molecules [35, 44].

TPA is a tumor promoter that induces high ROS, especially O_2_^−^, production [28] and therefore it is often used to characterize the effect of superoxide dismutases in vitro [45] and in vivo models [46]. In the current study TPA administration time-dependently increased redox gene expressions (Fig. 5) but dose-dependently showed differential response in redox gene expression (Fig. 6). Because *sod3* mRNA dose-dependent response to TPA (Fig. 6e) suggested similar expression pattern as observed in FRLT5 *H*-*RasV12* clones V13, V21, and V39 (Fig. 4f), we characterized in more detail the effect of *sod3* over-expression in TPA treated PC Cl3 cells (Fig. 7). Our previous observations have suggested a coordinated action of SOD3 and RAS small GPTase signal transduction thus indicating that SOD3 supports growth by increasing RAS-ERK1/2 and PI3 K-AKT signal transduction pathway activation [8, 31, 47, 48]. The current data suggesting increased cell proliferation in TPA treated cells in the presence of *SOD3* as compared to control cells (Fig. 7) supports previous observations suggesting that SOD3 may be a growth promoter contributing to cell proliferation at early phase of tumorigenesis.

## Conclusions

As a conclusion, according to our data PC Cl3, PC PTC1, PC E1A cell model systems corresponded to human *NOX2*, *NOX4*, *SOD3*, *CATALASE*, *GPX5*, and *GPX7* gene expressions in thyroid cancer. Used FRLT5 clones V13, V21, and V39 modeled human *NOX2*, *NOX4*, *SOD2*, *SOD3*, *CATALASE*, *GPX1*, *GPX2*, *PRDX2*, and *PRDX3* gene expressions. TPA administration to rat thyroid PC Cl3 cells could be used to study the effect of *NOX2*, *NOX4*, *SOD2*, *SOD3*, *CATALASE*, *GPX1*, *GPX2*, and *PRDX4* genes.

## Methods

### Cells

Rat thyroid PC Cl3, PC PTC1, PC E1A, FRLT5, and FRTL5-related cell clones V13, V21, and V39 stably transfected with *H*-*RasV12* expression plasmid [2] were cultured in Ham’s F12 medium Coon’s modified (Sigma, St Louis, MO, USA) supplemented with 5 % calf serum (Life Technologies, Inc., Carlsbad, CA, USA), penicillin (100 U/mL) (Sigma), and streptomycin (100 mg/L) (Sigma). PC Cl3 and FRLT5 cells (modeling normal thyroid cells) were additionally supplemented with 10 nM thyroid stimulating hormone (TSH), 10 nM hydrocortisone, 100 nM insulin, 5 mg/mL transferrin, 5 nM somatostatin, and 20 mg/mL glycyl-histidyl-lysine.

### Treatment of cells

To study effect of TSH on redox gene expression, FRLT5 cells were cultured 3 days without hormone supplementation, stimulated for 6 h with 10 nM TSH, and collected for RNA isolation. To study dose-dependent effect of tetradecanoylphorbol acetate (TPA) (Sigma) on redox gene expression and cellular growth, PC Cl3 cells were treated with 0.016, 1.6 or 16 nM TPA for 8 h and pelleted for RNA isolation. To study time-dependent effect of TPA on redox gene expression, PC Cl3 cells were supplemented with fresh medium containing 1.6 nM TPA at day 0, day 1, and day 2. Cells were collected on day 1, day 2, and day 3 for RNA isolation. To study effect of TPA on cell growth, PC Cl3 control cells and PC Cl3 cells over-exressing *SOD3* (kindly provided by professor Stefan L. Marklund from the University of Umeå, Sweden) were treated with 1.6 and 16 nM TPA and prepared for BrdU DNA replication analysis, growth curve analysis, and microscopy. *SOD3* over-expressing and control plasmid PC Cl3 cells were prepared by transfecting cells with Fugene 6 (Roche, Mannheim, Germany) followed by geneticin antibiotic (Sigma) selection to create stable cells lines.

### Real time PCR for mRNA expression analysis

For mRNA expression total RNA was extracted using an RNeasy mini kit (Qiagen, Hilden, Germany). RNA (1 μg) was reverse transcribed with Quantitect Reverse^®^ Transcription Kit (Qiagen). Primers were designed with (http://www-genome.wi.mit.edu/cgi-bin/primer/primer3_www.cgi). cDNA was done by GeneAmp RNA PCR Core Kit system (Qiagen). Reactions were performed in triplicate using SYBR Green PCR Master mix (Applied Biosystems, Foster City, CA, USA) and iCycler (BioRad, Hercules, CA, USA). Fluorescent threshold values were calculated using formula 2^−(sample 1 ΔCt − sample 2 ΔCt)^, where ΔCt is difference between amplification fluorescent threshold (Ct) of mRNA of interest, and Ct of *ß*-*actin* mRNA used as housekeeping gene. Primers are listed in Additional file 1: Table S1.

### Growth analysis

For BrdU DNA replication analysis PC Cl3 cell cultures grown on coverslips were supplemented with 10 mM bromodeoxyuridine (BrdU; Roche, Basel, Switzerland) for 15 min. Subsequently, cells were fixed in 3 % paraformaldehyde (Sigma) and permeabilized with 0.2 % Triton X-100 (Sigma). The coverslips were incubated with anti-BrdU antibody followed by FITC–conjugated secondary antibody (Jackson ImmunoResearch Laboratories Inc., West Grove, PA, USA). Nuclei were counterstained with HOECST (Sigma). For growth curve analysis PC Cl3 cells were grown in 6-well dishes. The number of cells was counted in triplicate from control and SOD3 wells in each analysis date by burker chamber using formula: number of cells/ml = average of cells in each square × 10^4^ × dilution factor.

### Statistical analyses

The experiments were repeated at least three times. All results are expressed as the mean ± SD. The p values (*p < 0.05, **p < 0.01, ***p < 0.001) were determined by two-tailed independent samples *t* test.

## Additional file

10.1186/s12935-015-0264-3 PCR primers.

## Abbreviations

cAMP: cyclic adenosine monophosphate
BRAF: serine threonine protein kinase
ERK: extracellular signal regulated kinase
GPX: glutathione peroxidase
H_2_O_2_: hydrogen peroxide
H-RasV12: Harvey sarcoma virus mutated
JNK: c-Jun terminal kinase
MAPK: mitogen activated protein kinase
MEK: mitogen activated protein kinase kinase
NOX: nicotinamide adenine dinucleotide phosphate-oxidase
PI3 K-AKT: phosphatidylinositol-4,5-bisphosphate 3-kinase-protein kinase B
PKA: protein kinase A
PKC: protein kinase C
PRDX: peroxiredoxin
REDOX: reduction oxidation
RET: ret proto-oncogene
ROS: reactive oxygen species
SOD: superoxide dismutase
O_2_^−^: superoxide anion
SRC: proto-oncogene tyrosine-protein kinase Src
TPA: tetradecanoylphorbol acetate
TSH: thyroid stimulating hormone
TSH-R: thyroid stimulating hormone receptor
